# Supplementary material for: Application of glutamate weighted CEST in brain imaging of nicotine dependent participants in vivo at 7T
Source: PLoS One. 2024 Feb 16;19(2):e0297310. doi: 10.1371/journal.pone.0297310 (PMC10871471; doi:10.1371/journal.pone.0297310)
Supplement: Attachment — Submitted filename: Response to Reviewers.docx [file pone.0297310.s003.docx]

**General Comments to All Reviewers:**

**Reviewer 1:**

**R.1.1** The abstract states, “T1-weighted images were used to segment the GluCEST images into seven cortical and four subcortical regions”. However, the methods/results only specify seven subcortical regions. Can you explain this?

**Our Response:** We have clarified this discrepancy between the abstract and the main text by stating that both general white and gray matter ROIs were used along with a further segmented into seven gray matter ROIs.

**R.1.2** What is the statistical significance of the negative age correlation found in one region?

**Our Response:** We performed a linear regression model on the correlation between age and the cingulate gyrus region for smokers and found that the correlation was statistically significant. We report this result in the main text.

**R.1.3** The binding of nicotine to the glutamate receptor should increase the glutamate concentration. So why would there be a negative age dependence in smokers?

**Our Response:** Based on previous literature, we have provided a potential explanation of the effect seen.

**R.1.4** Discussion, 2^nd^ paragraph “were observed” done twice.

**Our Response:** Done

**R.1.5** How about other contributions to the GluCEST signal? Could these be a limitation?

**Our Response:** We mention some of the technical limitations (B_1_ and B_0­_ inhomogeneities as well as partial voluming) that may have confounded the GluCEST signal and affected the results.

**Reviewer 2:**

**R.2.1** Analysis of conflicting literature: In the context of this research, the broader literature on regional glutamate differences in smokers versus non-smokers presents conflicting findings. The authors should delve into potential factors contributing to these discrepancies and outline their proposed solutions. Notably, previous studies were conducted at 4T field strengths and below, utilizing various MRS techniques. While GluCEST imaging offers simultaneous measurement advantages, the authors should acknowledge the limitations and potential confounding factors, such as variability of ROI definitions, total intracranial volume, stability of measurements and gender differences.

**Our Response:** We agree and have added additional information regarding the limiting factors of previous studies that have been performed.

**R.2.2** Sample Size Justification: The authors need to elaborate on their rationale for selecting a relatively small sample size. A thorough discussion on how this choice may impact the robustness of their conclusions is essential. Additionally, addressing whether the sample size aligns with statistical power considerations would contribute to the overall validity of the study.

**Our Response:** Due to the scope of this study being only an initial pilot study, the resources available to recruit subjects were limited. We acknowledge this as a limitation of the study and a future direction of the project.

**R.2.3** Consideration of Additional Biological Differences: Beyond glutamate levels, the authors should explore other quantitatively measurable biological differences between smokers and non-smokers, such as gray matter volume and nicotine concentrations. Including these factors as covariates in the statistical analysis could provide a more comprehensive understanding of the observed GluCEST imaging results.

**Our Response:** We performed an additional analysis based on the gray and white matter volumes to the average GluCEST contrast and present those additional results in the main text and as Figure 5.

**R.2.4** Detailed Methodological Information: Provide more comprehensive details on tissue segmentation, ROI definition and size, and the positioning of GluCEST imaging slices. In particular, clarify how the authors standardized the definition of the same oblique axial slice across different subjects. This information is crucial for replicability and understanding the precision of the methodology employed.

**Our Response:** We provide additional details on the segmentation ROI method used and the placement of the imaging slice.

**R.2.5** Proofreading and Terminology: Conduct a thorough proofread to correct any grammatical errors and spelling mistakes, such as ‘brian’ instead of ‘brain’. Ensuring precise and accurate terminology is used throughout the paper contributes to the overall professionalism and credibility of the research.

**Our Response:** Done
